# Persistent Shock After Successful PCI Due to CPR-Related Internal Mammary Artery Hemorrhage

**DOI:** 10.1016/j.jaccas.2026.107728

**Published:** 2026-03-16

**Authors:** Ashley M. Tate, Alejandro Pinedo, Samantha Mutai, Darren C. Tsang, Cayce S. Workman, Shannon X. Li, Steve Attanasio

**Affiliations:** a Division of Cardiology, Department of Internal Medicine, Rush University Medical Center, Chicago, Illinois, USA; b Department of Vascular and Interventional Radiology, Rush University Medical Center, Chicago, Illinois, USA

**Keywords:** acute coronary syndrome, complication, coronary angiography, myocardial infarction, percutaneous coronary intervention

## Abstract

**Background:**

Shock is a common complication of acute myocardial infarction and cardiac arrest. Internal mammary artery (IMA) injury is a rare cause of post–cardiopulmonary resuscitation (CPR) hemorrhagic shock.

**Case Summary:**

An 80-year-old woman presenting with inferior ST-segment elevation myocardial infarction underwent percutaneous coronary intervention to the left circumflex artery with intra-aortic balloon pump support after cardiac arrest treated with mechanical CPR. Persistent hypotension prompted computed tomography angiography, revealing a mediastinal hematoma with active right IMA extravasation. Urgent embolization resulted in hemodynamic stabilization and clinical recovery.

**Discussion:**

This case illustrates the diagnostic challenge of persistent shock after coronary revascularization when hemodynamic instability is disproportionate to the cardiogenic component and highlights CPR-related IMA injury as a rare but life-threatening extracardiac cause.

**Take-Home Message:**

Persistent hypotension after ST-segment elevation myocardial infarction revascularization should prompt evaluation for extracardiac causes of shock, including CPR-related IMA injury, a rare complication that is amenable to prompt embolization.

## Visual Summary

**Figure:**
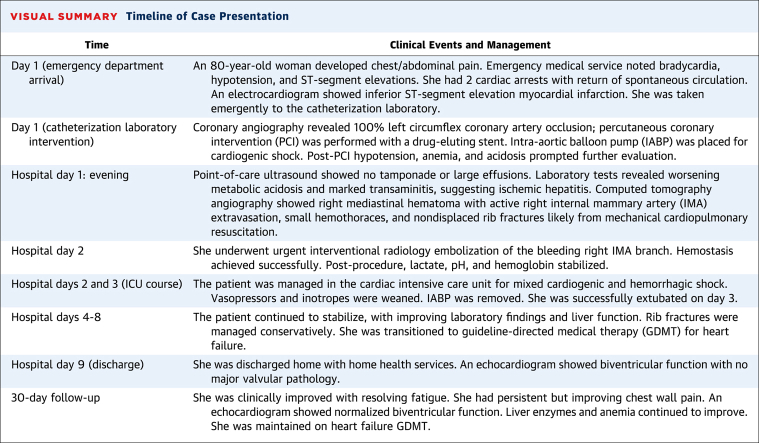

## History of Presentation

An 80-year-old woman with no prior cardiac history experienced acute-onset chest and abdominal pain approximately 30 minutes before emergency medical services arrival. She was bradycardic and hypotensive with ST-segment elevations on electrocardiogram. Intravenous atropine was administered, and transcutaneous pacing was initiated en route. On arrival, she was obtunded and rapidly became pulseless. Cardiopulmonary resuscitation (CPR) and advanced cardiac life support were initiated, with return of spontaneous circulation after 1 cycle of chest compressions using a mechanical device (LUCAS, Stryker Medical) and 1 mg of intravenous epinephrine.

**Take-Home Messages**

• Persistent hypotension after otherwise successful PCI should prompt evaluation for extracardiac etiologies of shock.
• Although rare, internal mammary artery injury is a potentially fatal complication associated with mechanical cardiopulmonary resuscitation and PCI that can mimic refractory cardiogenic shock; this complication requires early detection by computed tomography angiography and definitive intervention by endovascular embolization.

**Figure 1:**
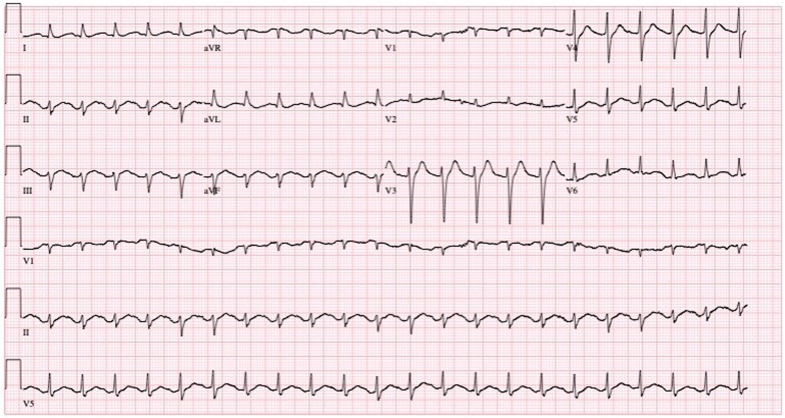
Post-Arrest Electrocardiogram Demonstrating Inferior ST-Segment Elevation Myocardial Infarction (II, III, and aVF) and ST-Segment Depressions in V_3_-V_4_

A postarrest electrocardiogram redemonstrated inferior ST-segment elevations (leads II, III, and aVF) with reciprocal ST-segment depressions in leads V_3_-V_4_ (Figure 1). The patient again became pulseless, requiring a second cycle of CPR and an additional 1 mg of intravenous epinephrine with return of spontaneous circulation. She was intubated for airway protection, and norepinephrine infusion was initiated for profound hypotension (blood pressure 50s/30s mm Hg). Postintubation physical examination revealed an ill-appearing woman with a Glasgow Coma Scale score of 3, tachycardia, and an otherwise unremarkable cardiovascular examination, without signs of trauma or peritonitis.

**Figure 2:**
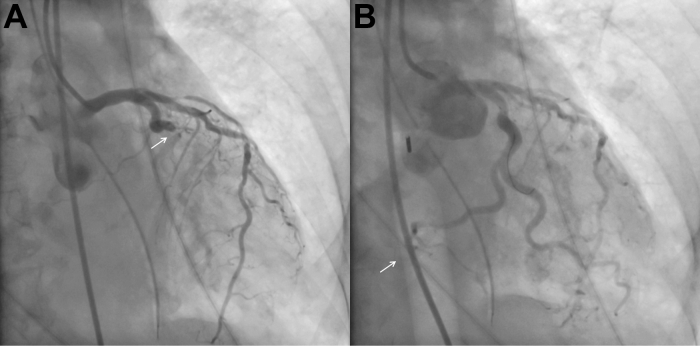
Emergent Cardiac Catheterization (A) Coronary angiography demonstrating acute total occlusion of the mid–left circumflex artery (white arrow). (B) Angiography after percutaneous coronary intervention and intra-aortic balloon pump placement (white arrow).

Point-of-care ultrasonography demonstrated severely reduced left ventricular systolic function with inferolateral wall hypokinesis. Emergent cardiac catheterization with coronary angiography via right femoral access revealed an acute thrombotic occlusion of a large, dominant left circumflex artery (Figure 2). An intra-aortic balloon pump (IABP) was placed for temporary mechanical circulatory support (tMCS), and percutaneous coronary intervention (PCI) was performed with deployment of a 4.0 × 22-mm drug-eluting stent, restoring brisk anterograde flow. Periprocedural unfractionated heparin was administered, and loading doses of aspirin (324 mg) and ticagrelor (180 mg) were given after orogastric tube placement. The patient was not receiving chronic anticoagulant or antiplatelet therapy before presentation. Despite successful revascularization, she had ongoing severe hypotension that was responsive to intravenous fluid and norepinephrine administration and was transferred to the cardiac intensive care unit at a tertiary center.

## Past Medical History

The patient's past medical history included colon cancer status post chemotherapy, rheumatoid arthritis, Sjögren syndrome treated with intermittent corticosteroids, diabetes mellitus, osteoporosis, and asthma. She had no known cardiovascular disease.

## Differential Diagnosis

The patient's initial presentation was consistent with Society for Cardiovascular Angiography and Interventions (SCAI) stage E cardiogenic shock due to acute myocardial infarction complicated by circulatory collapse. Persistent hypotension despite revascularization and tMCS with IABP broadened the differential to include ongoing SCAI stage D cardiogenic shock from severe myocardial dysfunction, right ventricular failure, acute severe mitral regurgitation, pericardial effusion with tamponade, retroperitoneal hemorrhage, hemothorax, tension pneumothorax, acute stent thrombosis, and distributive shock from concomitant sepsis, adrenal insufficiency, or devastating neurologic injury.

## Investigations

Initial laboratory evaluation revealed elevated high-sensitivity troponin (251.4 ng/L), arterial pH of 7.245, bicarbonate of 18 mEq/L, total carbon dioxide of 19 mEq/L, arterial lactate of 2.7 mmol/L, and elevated aspartate aminotransferase (AST) 121 U/L and alanine aminotransferase (ALT) 86 U/L, consistent with acute myocardial injury and systemic hypoperfusion.

Immediately after salvage PCI with tMCS, point-of-care venous blood gas analysis demonstrated worsening metabolic acidosis with inadequate respiratory compensation (pH 7.140; Pco_2_ 47 mm Hg; bicarbonate 16 mEq/L), and arterial lactate 2.3 mmol/L consistent with persistent shock. A pulmonary artery catheter was placed, demonstrating low right-sided filling pressures (right atrium 3 mm Hg) and normal pulmonary artery pressures (19/12 mm Hg; mean 13 mm Hg). Mixed venous O_2_ saturation was 67%, with an assumed Fick cardiac output and index of 3.7 L/min and 2.6 L/min/m^2^, respectively. Point-of-care ultrasonography revealed small right-sided cardiac chambers, a collapsible inferior vena cava, and no significant mitral regurgitation, pericardial effusion, intraabdominal free fluid, pleural effusion, or pneumothorax. A multidisciplinary shock team determined that the patient was unlikely to benefit from tMCS upgrade, and she was deemed not a candidate for extracorporeal membrane oxygenation.

**Figure 3:**
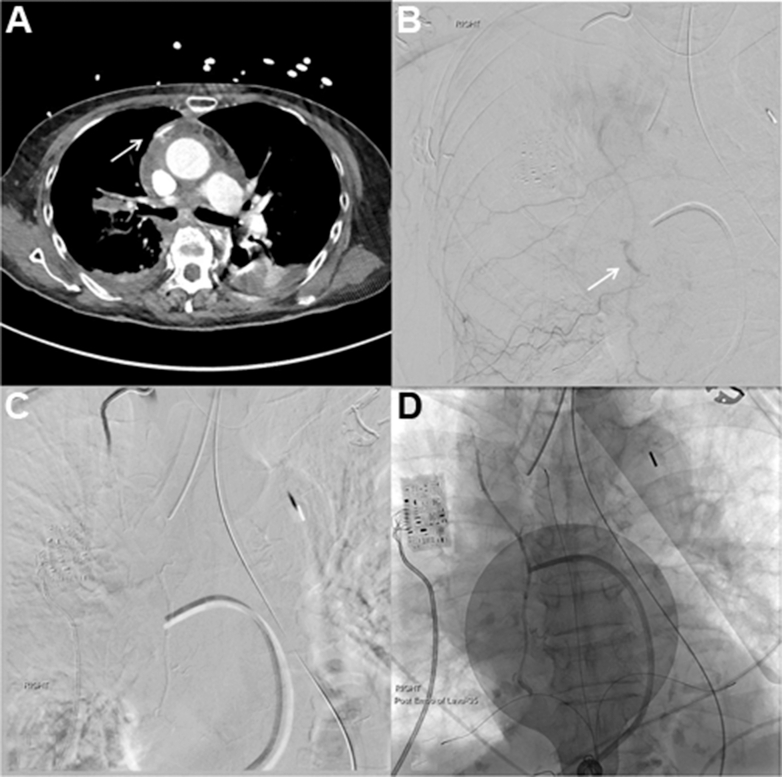
Right Internal Mammary Artery Hemorrhage (A) Computed tomography angiography demonstrating contrast pooling (white arrow) in the anterior mediastinum consistent with active extravasation. (B) Selective right internal mammary digital subtraction angiogram confirming the area of extravasation (white arrow). (C) Digital subtraction angiogram after embolization with ethylene vinyl alcohol copolymer (LAVA Liquid Embolic System; Sirtex) confirming cessation of extravasation. (D) Postembolization (anterior-posterior) fluorogram showing the LAVA cast within the right internal mammary artery.

In the cardiac intensive care unit, empiric broad-spectrum antibiotics and stress-dose corticosteroids were initiated for possible septic shock and adrenal insufficiency, respectively. Thermodilution cardiac output and index were 2.6 L/min and 1.7 L/min/m^2^, respectively, despite 1:1 IABP support, prompting initiation of dobutamine at 5 μg/kg/min. Laboratory testing revealed persistent acidemia with a mixed metabolic and respiratory disturbance (arterial pH 7.209; Pco_2_ 49 mm Hg; lactate 1.9 mmol/L). Liver chemistries increased markedly (AST 853 U/L; ALT 482 U/L; international normalized ratio 1.26), concerning for ischemic hepatitis. Serial hemoglobin measurements after PCI demonstrated an early and progressive decline from 11.7 g/dL on presentation to 8.9 g/dL within hours of arrival to the cardiac intensive care unit, prompting transfusion of one unit of packed red blood cells. In the absence of overt procedural blood loss, femoral access-site bleeding, or evidence of intraabdominal free fluid on bedside ultrasound, this decline prompted cross-sectional imaging. Computed tomography angiography of the chest, abdomen, and pelvis with intravenous contrast demonstrated a right upper mediastinal hematoma with active extravasation from the right internal mammary artery (IMA), small bilateral hyperdense pleural effusions concerning for hemothoraces, multiple bilateral nondisplaced rib fractures, and no retroperitoneal hemorrhage (Figure 3A).

## Management

Emergent embolization was performed by interventional radiology. Right radial access was selected owing to the presence of an IABP. Because of challenging arterial takeoff, a narrow-radius reverse-curve catheter (Hawkins Hairpin; AngioDynamics) was required to engage the origin of the IMA. Angiography confirmed active extravasation from a mid–right IMA branch (Figure 3B). Given recent antiplatelet therapy loading after PCI, a liquid embolic agent (ethylene vinyl alcohol copolymer; LAVA Liquid Embolic System; Sirtex) was selected, as platelet activation is not required for occlusion. No immediate complications were observed (Figures 3C and 3D). Thoracic surgery was consulted and recommended conservative management of rib fractures.

Post-procedure, acid-base status normalized (arterial pH 7.398), hemoglobin stabilized (11.3 g/dL), and tissue perfusion improved, as evidenced by reduced lactate levels (2.3-1.5 mmol/L) and liver enzymes (AST 559 U/L; ALT 332 U/L), consistent with resolution of cardiogenic and hemorrhagic shock.

## Outcome and Follow-Up

The hospital course was complicated by hemoptysis secondary to pulmonary contusions, aspiration pneumonia, and pain control challenges. Postembolization transthoracic echocardiography demonstrated mildly reduced left ventricular ejection fraction (40%-45%), mild right ventricle dilation and dysfunction, and no significant valvular abnormalities. Vasopressors and inotropes were discontinued within 48 hours, and the patient was extubated on hospital day 3 after IABP removal. She was discharged on hospital day 9 and remained clinically stable at follow-up with normalization of biventricular function on guideline-directed medical therapy.

## Discussion

Emergent PCI remains first-line therapy for ST-segment elevation myocardial infarction, particularly in the setting of cardiogenic shock, and was appropriately pursued in this patient in accordance with contemporary American College of Cardiology/American Heart Association guidelines.^1^ The SCAI shock classification provides a useful framework for risk stratification and guiding escalation of tMCS after PCI, integrating both clinical and hemodynamic parameters allowing for reassessment over time.^2^

The patient initially met criteria for SCAI stage E cardiogenic shock, given cardiac arrest requiring CPR and vasopressor support. Invasive hemodynamics after revascularization demonstrated ongoing cardiogenic shock despite IABP support, with a thermodilution cardiac index of 1.7 L/min/m^2^, consistent with SCAI stage D physiology. However, low right-sided filling pressures, normal pulmonary artery pressures, and marked volume responsiveness were discordant with isolated refractory cardiogenic shock, suggesting an additional extracardiac contributor to the patient's instability.

This discordance prompted further evaluation, ultimately revealing a hemorrhagic source: right IMA laceration. The patient underwent femoral-access PCI without instrumentation near the IMA, implicating blunt thoracic trauma from CPR as the likely mechanism of injury. Prior reports demonstrate that CPR-related IMA injury may occur even in the absence of overt chest wall trauma, underscoring the need for a high index of suspicion in unstable patients with otherwise unexplained anemia after resuscitation.^3^^,^^4^ Early physical examination, chest radiography, and bedside ultrasonography in this case were unrevealing, and the diagnosis emerged only after recognition of an unexplained hemoglobin decline and shock physiology inconsistent with cardiogenic shock alone.

Notably, this presentation occurred in the setting of acute coronary syndrome, where emergent antithrombotic therapy may exacerbate hemorrhage and obscure its recognition. In contrast to many previously reported cases, this patient was not receiving chronic antithrombotic therapy before presentation, emphasizing that bleeding risk arose acutely in the context of CPR and acute coronary syndrome management. Diagnostic complexity was further heightened by IABP support. Similar to extracorporeal membrane oxygenation described in prior reports, tMCS has been associated with delayed recognition of CPR-related vascular injury, potentially obscuring hemorrhagic shock in post–cardiac arrest patients.^5^^,^^6^ In such cases, reliance on serial hemoglobin trends, filling pressures, and volume responsiveness is essential, and prior case series support a low threshold for cross-sectional imaging when shock physiology is discordant.^5^^,^^6^ Computed tomography angiography is the diagnostic modality of choice for detecting thoracic arterial extravasation,^7^ rapidly identifying the source of hemorrhage while excluding other causes of post-PCI shock. Prompt endovascular embolization resulted in rapid stabilization and ensured this patient's survival.

## Conclusions

This case highlights the importance of considering CPR-related vascular injury in patients with shock after PCI, particularly in patients receiving antithrombotic therapy. Hemorrhagic shock from IMA injury initially mimicked persistent cardiogenic shock. Recognition of this patient's disproportionate hemodynamic collapse prompted timely diagnostic imaging and endovascular intervention, resulting in rapid stabilization.

## Funding Support and Author Disclosures

The authors have reported that they have no relationships relevant to the contents of this paper to disclose.

**Visual Summary:** Timeline of Case Presentation

Time	Clinical Events and Management
Day 1 (emergency department arrival)	An 80-year-old woman developed chest/abdominal pain. Emergency medical service noted bradycardia, hypotension, and ST-segment elevations. She had 2 cardiac arrests with return of spontaneous circulation. An electrocardiogram showed inferior ST-segment elevation myocardial infarction. She was taken emergently to the catheterization laboratory.
Day 1 (catheterization laboratory intervention)	Coronary angiography revealed 100% left circumflex coronary artery occlusion; percutaneous coronary intervention (PCI) was performed with a drug-eluting stent. Intra-aortic balloon pump (IABP) was placed for cardiogenic shock. Post-PCI hypotension, anemia, and acidosis prompted further evaluation.
Hospital day 1: evening	Point-of-care ultrasound showed no tamponade or large effusions. Laboratory tests revealed worsening metabolic acidosis and marked transaminitis, suggesting ischemic hepatitis. Computed tomography angiography showed right mediastinal hematoma with active right internal mammary artery (IMA) extravasation, small hemothoraces, and nondisplaced rib fractures likely from mechanical cardiopulmonary resuscitation.
Hospital day 2	She underwent urgent interventional radiology embolization of the bleeding right IMA branch. Hemostasis achieved successfully. Post-procedure, lactate, pH, and hemoglobin stabilized.
Hospital days 2 and 3 (ICU course)	The patient was managed in the cardiac intensive care unit for mixed cardiogenic and hemorrhagic shock. Vasopressors and inotropes were weaned. IABP was removed. She was successfully extubated on day 3.
Hospital days 4-8	The patient continued to stabilize, with improving laboratory findings and liver function. Rib fractures were managed conservatively. She was transitioned to guideline-directed medical therapy (GDMT) for heart failure.
Hospital day 9 (discharge)	She was discharged home with home health services. An echocardiogram showed biventricular function with no major valvular pathology.
30-day follow-up	She was clinically improved with resolving fatigue. She had persistent but improving chest wall pain. An echocardiogram showed normalized biventricular function. Liver enzymes and anemia continued to improve. She was maintained on heart failure GDMT.

**Equipment List:** 

Salvage Percutaneous Coronary Intervention	Emergent Right Internal Mammary Artery Embolization
Imaging • Fluoroscopy system • Eagle Eye Platinum Digital Intravascular Ultrasound catheter (Philips) Vascular access • Ultrasound machine (Philips) • Micropuncture introducer set, 5-F × 10 cm (Cook Medical) • Ultimum Hemostasis Introducer sheath, 6-F × 12 cm (Abbott) • GuideRight 3-mm J-tip wire, 0.035 inch × 150 cm (Abbott) • Emerald Guidewire 3-mm J-tip wire, 0.035 inch × 260 cm (Cordis) Diagnostic and guide catheters • Performa JL 4.0, 6-F × 100 cm (Merit Medical) • Launcher JR 4.0, 6-F × 100 cm (Medtronic) • Launcher EBU 3.5, 6-F × 100 cm (Medtronic) Temporary mechanical circulatory support • Avanti Sheath Introducer, 8-F × 11 cm (Cordis) • Sensation Intra-aortic balloon pump, 7-F × 34 cc (Getinge) • ProCare Knee Immobilizer, 20 inches (McKesson) Intervention • Hi-Torque balanced middleweight guidewire, 0.014 inch × 190 cm (Abbott) • Euphora semicompliant balloon catheter, 2.5 × 15 mm (Medtronic) • Onyx Frontier zotarolimus-eluting stent, 4.0 × 22 mm (Medtronic) • NC Euphora noncompliant balloon catheter, 4.5 × 8 mm (Medtronic) • Angio-Seal VIP, 6-F (Terumo Medical Corporation) Pulmonary artery catheter • Arrow Introducer Sheath Kit, 8.5-F (Arrow Medical) • Swan Ganz Thermodilution VIP, 7.5-F (Edwards Lifesciences)	Imaging • Fluoroscopy and digital subtraction angiography system Vascular access • Ultrasound machine (Philips) • Micropuncture Introducer Set Pediatric, 4-F × 10 cm (Cook Medical) • GlideSheath Slender, 5-F (Terumo Medical Corporation) • TR Band Regular (Terumo Medical Corporation) Guidewires • Nitrex Guidewire, 0.018 inch × 80 cm (Cook Medical) • Bentson Wire Guide, 0.035 inch × 180 cm (Cook Medical) • Synchro Select Soft, 0.014 in × 215 cm (Stryker) • Angled Glidewire, 0.035 inch × 180 cm (Terumo Medical Corporation) • Glidewire Angled Exchange, 0.035 inch × 260 cm (Terumo Medical Corporation) • Guidwire GT Double Angle, 0.018 inch × 180 cm (Terumo Medical Corporation) • Amplatz Super Stiff Straight Tip, 0.035 inch × 180 cm (Boston Scientific) • Rosen Curved Wire Guide, 0.035 inch × 180 cm (Cook Medical) • Glidewire Stiff Angled, 0.035 inch × 180 cm (Terumo Medical Corporation) Diagnostic and guide catheters • Glidecath Nontaper Angle, 4-F × 65 cm (Terumo Medical Corporation) • Mariner Hawkins Hairpin catheter, 5-F, 90 cm (AngioDynamics) • Progreat Microcatheter, 2.8-F × 150 cm (Terumo Medical Corporation) Embolic agent • LAVA Liquid Embolic System, Ethylene vinyl alcohol copolymer liquid embolic agent (Sirtex)
